# Pseudoboehmite as a drug delivery system for acyclovir

**DOI:** 10.1038/s41598-021-94325-y

**Published:** 2021-07-29

**Authors:** Renato Meneghetti Peres, Jéssica Maiara Leme Sousa, Mariana Oliva de Oliveira, Maura Vincenza Rossi, Rene Ramos de Oliveira, Nelson Batista de Lima, Ayrton Bernussi, Juliusz Warzywoda, Bruno Sarmento, Antonio Hortencio Munhoz

**Affiliations:** 1 School of Engineering, Mackenzie Presbyterian University, Rua da Consolação, 930, Building 33, Consolação, São Paulo, SP 01302-907 Brazil; 2 CCBS, Mackenzie Presbyterian University, São Paulo, Brazil; 3 IPEN, São Paulo, SP Brazil; 4 Department of Electrical and Computer Engineering and Nano Tech Center, Texas Tech University, Lubbock, TX 79409 USA; 5 Materials Characterization Center, Whitacre College of Engineering, Texas Tech University, Lubbock, TX 79409 USA; 6 INEB-Instituto de Engenharia Biomédica and i3S-Instituto de Investigação e Inovação em Saúde Universidade do Porto, Porto, Portugal

**Keywords:** Biomaterials, Nanoscale materials

## Abstract

Herpes simplex virus is among the most prevalent sexually transmitted infections. Acyclovir is a potent, selective inhibitor of herpes viruses and it is indicated for the treatment and management of recurrent cold sores on the lips and face, genital herpes, among other diseases. The problem of the oral bioavailability of acyclovir is limited because of the low permeability across the gastrointestinal membrane. The use of nanoparticles of pseudoboehmite as a drug delivery system in vitro assays is a promising approach to further the permeability of acyclovir release. Here we report the synthesis of high purity pseudoboehmite from aluminium nitrate and ammonium hydroxide containing nanoparticles, using the sol–gel method, as a drug delivery system to improve the systemic bioavailability of acyclovir. The presence of pseudoboehmite nanoparticles were verified by infrared spectroscopy, transmission electron microscopy, and X-ray diffraction techniques. In vivo tests were performed with Wistar rats to compare the release of acyclovir, with and without the addition of pseudoboehmite. The administration of acyclovir with the addition of pseudoboehmite increased the drug content by 4.6 times in the plasma of Wistar rats after 4 h administration. We determined that the toxicity of pseudoboehmite is low up to 10 mg/mL, in gel and the dried pseudoboehmite nanoparticles.

## Introduction

The global incidence of herpes simplex virus infections (HSV-1 and HSV-2) have risen worldwide and more than one-third of the world’s population has been already affected. A situation is further complicated because most persons with genital HSV infection have not received a diagnosis and herpes infected patients are more susceptible to HIV infections. The HSV infection is more common in women than in men^1^ and according to Klysik et al.^2^, 50–90% of adults are seropositive for HSV-1 and HSV-2 worldwide.

In 2008, in the United States, an estimated 110 million men and women were infected with sexually transmitted diseases^3^. Of these, approximately 20% of infections (22.1 million) occurred among young women and men aged 15–24 years^3^. The herpes virus is resistant to treatment and once the HSV virus enters the human body, it cannot be completely eradicated because HSV viruses can change into their latent form and therefore survive the treatment. The ocular infection, for example, is the major cause of corneal blindness in the Western World^2^. Clinical studies have also demonstrated that allogeneic hematopoietic cell transplantation recipients frequently experience complications from varicella-zoster virus infection. It has been shown recently that continuous acyclovir administration could be the best option to treat severe HSV-1 infectious patients^4,5^. In a recent study, it has been suggested a relation between Herpesviruses and Alzheimer's disease^2^. This makes acyclovir a prospectively important drug in the treatment of Alzheimer's disease. However, there is no consensus for the treatment of Alzheimer's disease until now and recent research associates *Herpes viridae* (*H-viridae*) in the pathogenesis of late-onset dementia. It was observed that the accumulation of β-amyloid in the human brain and peripheral tissues of patients with late-onset Alzheimer's disease occurred^6^.

In a transgenic mouse model (5XFAD) the infection with *H-viridae* begins β-amyloid plaque deposition, and this is also observed in the brain of patients with late-onset Alzheimer's disease^7^. Wozniak et al. suggest that herpes simplex virus type 1 is a strong risk factor for Alzheimer's disease^8^. Hui et al. observed that the combination of acyclovir and dexamethasone could protect against Alzheimer's disease-causing β-amyloid oligomer-induced spatial cognitive impairments. They also observed that acyclovir or dexamethasone alone does not give the same results^9^. Piper et al. reported the use of acyclovir in glioblastoma treatment. In three patients with a diagnosis of viral encephalitis due to symptoms and radiographic results, it was observed clinical improvement by the use of acyclovir. But later, the diagnosis concludes that there was glioblastoma in the same region^10^.

A major issue confronting the use of acyclovir for herpes virus treatment is its low permeability across the gastrointestinal membrane. In order to enhance the acyclovir permeability, pseudoboehmite nanoparticles can be used as a delivering system. Pseudoboehmite (i.e., a poorly crystallized boehmite) is a synthetic aluminium compound that can be produced by sol–gel. One important property of pure pseudoboehmite is its high absorption capacity. The overall improvement of acyclovir after oral administration relies on the capacity of Pseudoboehmite in increasing the solubility of the drug due to the sustained release. By that, the drug is retained longer in the absorption window, compared to the insoluble drug, rapidly removed from the GIT.

Pseudoboehmite has a similar structure to boehmite and this was verified by comparing X-ray diffraction patterns of boehmite and pseudoboehmite which exhibited nearly identical peak positions^11^. There is only a small difference in the pseudoboehmite crystal structure due to higher water incorporation resulting in a slightly larger unit cell than that of the boehmite. Pseudoboehmite can produce transition alumina like boehmite. The calcination at 500 °C transform it in gamma-alumina^15^. All transition alumina is synthetic^12–14^. Pseudoboehmite transforms to transition alumina according to Eq. (1)^15^.

1 $$ {\text{pseudoboehmite}} \to \gamma {\text{-alumina}} \to \delta {\text{-alumina}} \to \theta {\text{-alumina}} \to \alpha {\text{-alumina}} $$

In vitro studies of pseudoboehmite for controlled drug delivery systems reported lately include atenolol^16^, Glucantime^®^^17^, acyclovir^18,19^, and DOX, a chemotherapeutic anticancer drug^20^. With acyclovir, it was observed in in vitro experiments that pseudoboehmite promoted acyclovir solubility in water^18^. With Glucantime^®^ and atenolol^16,17^, the drug release was kept constant and there was no reaction of the drugs with pseudoboehmite. In DOX release, a mesoporous pseudoboehmite and upconversion nanoparticles (UCNPs), and the obtained nanocomposite (UCNPs-Al) could be performed as efficient transport of the drug into the cancer cell and release DOX from UCNPs-Al triggering by the mildly acidic environment. The laser scanning upconversion luminescence (UCL) imaging from UCNPs and the red fluorescence of DOX allow the nanocomposite to be applied to the cell imaging system simultaneously^20^.

Until now, no published research using nanoparticles of pseudoboehmite as a drug-delivering system in animals have been reported. This is the main contribution of this paper.

Acyclovir is an efficient drug for the treatment of herpes simplex types I and II, which acts against varicella-zoster, but the oral bioavailability is limited due to incomplete absorption. When this drug is available at a high dose of 800 mg it falls under class IV in the “the Biopharmaceutics Classification System” (BCS) and this classification means Low solubility low permeability drugs^21^. In a recent study using magnetite for acyclovir release^22^, when acyclovir is administered orally, it is partially absorbed from the gastrointestinal tract and approximately only 15–30 wt% of the dose is absorbed. The maximum plasma concentrations are reached in 1–2 h, and administration is usually required twice daily^23^.

Previous reports describe improvements of acyclovir administration using other methods. The use of biodegradable surfactant, Brij97^24^, improved the amount of permeated acyclovir^24^. Sadat concluded^22^ that there are different adsorption mechanisms for acyclovir on magnetite nanoparticles of different sizes. A matrix-based antiherpetic ring composed of poly(ethylene-*co*-vinyl acetate) was used to release acyclovir to the vaginal epithelium^25^ and New strategies based on complex nanostructures for developing advanced functional materials providing sustained release acyclovir were published recently using core–shell nanofiber^26^ and sericin hydrogel^27^.

The low permeability of acyclovir across the gastrointestinal membrane is a problem for oral bioavailability. The purpose of this study is the use of pseudoboehmite nanoparticles as a drug delivery system to improve the systemic bioavailability of acyclovir. The pseudoboehmite toxicity in living organisms proved experimentally non-toxic in Wistar rats’ studies^28^. Due to the difficulty of acyclovir release and the high incidence of herpes simplex virus infections in the world population, the study of improving acyclovir release is important and timely. Considering the association of the Herpes viruses and Alzheimer's disease the importance of acyclovir increases as a prospective drug for Alzheimer's disease treatment. In this work, the use of a pseudoboehmite in vivo drug release system to improve acyclovir release with Wistar rats was investigated.

## Experimental

The pseudoboehmite gel was synthesized via a sol–gel process using aluminium nitrate monohydrate, Al(NO_3_)_3_·9H_2_O (950 g/L), and ammonium hydroxide, NH_4_OH (28 NH_3_ wt%). Moroz et al.^13^ and Munhoz et al.^29^ have reported the use of these reagents as precursors of pseudoboehmite synthesis. The aluminium nitrate solution was dropped over an ammonium hydroxide solution with constant stirring to obtain a gel. The gel was vacuum filtered in a Buchner funnel and dried to obtain a powder by freeze-drying.

### Characterization of pseudoboehmite

The differential thermal analysis (DTA) and thermogravimetric analysis (TG) were performed using the Netzsch equipment, model STA 449F3 Jupiter. The sample was heated from room temperature to 1300 °C at 20°C min^−1^ rate under a dynamic N_2_ atmosphere (flow rate: 50 mL min^−1^). A quantity of 0.013 g of sample was placed in an open alumina crucible and the DTA-TG measurements were carried out simultaneously. The DTA and the TG were also used for the characterization of the pure acyclovir and the acyclovir pseudoboehmite sample.

Samples were imaged under different magnifications using a JEOL scanning electron microscope, model JSM-6510. Scanning electron microscopy (SEM) was performed using a secondary electron detector and energy-dispersive spectroscopy (EDS) detector. The sample was placed on a holder with the aid of a carbon double-face tape and coated with gold using an Edwards Sputter Coater model S150B.

X-ray diffraction analysis was performed using a Rigaku MultiFlex diffractometer with a monochromator, operating at 20 kV voltage, 40 mA current, scanning angle (2θ) ranging from 3° to 80°, Kα copper radiation (λ = 1.5418 Å), and a scanning speed of 0.015°s^−1^.

Pseudoboehmite was heated for 24 h. at 100 °C prior to nitrogen adsorption measurement^30–32^. The pseudoboehmite sample was used to obtain the nitrogen adsorption isotherm and to evaluate its specific surface area. The nitrogen adsorption isotherm was realized using BEL equipment, model “Belsorp Max”. The specific surface area of pseudoboehmite was determined by a multipoint Brunauer–Emmett–Teller (BET) method using the relative pressure (P/P_o_) range of 0.05–0.3^30–32^. The BET method consists of adsorption of inert gas (N_2_ in this case) at cryogenic temperatures, 77 K.

Infrared spectroscopy was performed in the 500–4000 cm^−1^ spectral region for samples dispersed in KBr pellets at a 1:300 ratio using the Shimadzu IRaffinity-1 FTIR equipment. The maximum optical power of this equipment is 0.1 mW.

Transmission electron microscopy (TEM) was realized using a Transmission Electron Microscope (TEM) Hitachi H-9500, from Materials Characterization Center, Whitacre College of Engineering, Texas Tech University. The samples were dispersed in ethanol using an ultrasound bath before supporting them in the copper grid covered with formvar.

Zeta potential was realized in a LitesizerTM Anton Paar equipment at 25 °C. 0.1 g of the pseudoboehmite gel was added in 100 mL distilled water and sonicated in ELMASONIC equipment for 30 min. After that, the zeta potential was determined.

### Evaluation of pseudoboehmite toxicity

The evaluation of the pseudoboehmite toxicity was realized for the pseudoboehmite gel and the dried pseudoboehmite.

Samples were prepared at 37 °C for 24 h in cell culture medium (DMEM + 10% FBS + 100 U/mL penicillin + 100 µg/mL streptomycin) at a concentration range of 0–10 mg/mL under orbital shaking (100 rpm). Controls were also submitted to the same procedure. Caco-2 cell line (ATCC) was used at passages 35–40 and maintained at 37 °C, 5% CO_2_ and 90% RH in culture medium (changed twice weekly). Cells were seeded in 96 well plates at 10,000 cells/well in 200 µL of culture medium and incubated for 24 h (37 °C/5% CO_2_/90% RH) before incubation with extracts of samples. Afterwards, nanoparticles were incubated with cells for 24 h (37 °C/5% CO_2_/90% RH). A negative control (culture medium) and a positive control (2% Triton X-100 in culture medium) were also tested. Afterwards, cells were washed trice with PBS pH 7.4 and incubated for 4 h with a culture medium containing 0.5 mg/mL of MTT. The culture medium was discarded and the formazan derivative crystals formed were dissolved using 200 µL of DMSO. Cell viability was assessed by measuring the absorbance at 570 nm (630 nm for background deduction) using a plate reader. Results were reported as viability percentage as compared to the negative control (100% viability), according to Eq. (2).

2 $$ \% \;{\text{viability }} = \, \left( {{\text{Abs}}\;570\;{\text{nm}}\;{\text{for}}\;{\text{sample/Abs}}\;570\;{\text{nm}}\;{\text{for}}\;{\text{negative}}\;{\text{control}}} \right) \, \times \, 100 $$

### Administration of acyclovir

The experiments with Wistar rats were carried out following the U.K.Animals (Scientific Procedures) Act, 1986 and associated guidelines, EU Directive 2010/63/EU for animal experiments. The Wistar rats used in the in vivo process were acquired from the University of São Paulo. They were kept in the vivarium in special humidity-controlled conditions at 21 ± 2 °C temperature and a 12/12 h light/dark cycle.

During these experiments, the animals were fed with commercial feed for rats, specific to their species (Nuvital brand), and water.

The maintenance, supply, treatment, and euthanasia of the animals followed the recommended ethical standards. In the oral administration procedure, from the initial time of administration to the time of metabolism of the drug, the animals were euthanized, free of pain or suffering, through an anaesthetic ketamine overload.

The dose of acyclovir and the acyclovir loaded pseudoboehmite nanoparticles were administered by gavage. For both administrations of the drug, the rats were divided into five groups each containing five Wistar rats. They were administered with 16.4 mg acyclovir /(kg Wistar rat) in 1 mL of distilled water. The drug was administered to 50 rats, each weighing approximately 330 g. Twenty-five rats were administered, e.g., 5.4 mg of acyclovir dissolved in 1 mL water. To the other twenty-five rats, it was administered a solution containing 5.4 mg of acyclovir supported in 100 mg of pseudoboehmite and 1 mL of water. Blood sampling was carried out at 1, 2, 3, 4, and 5 h after drug administration.

The pseudoboehmite was dispersed in water with the aid of stirring and ultrasound in ELMASONIC equipment for 30 min. After that, the acyclovir was added, and the mixture was homogenized at high speed for 2 min to promote the adsorption of the drug in the nanoparticles. The proportion used was 1 mL of water for 100 mg pseudoboehmite and 5.4 mg acyclovir.

After the administration of the drug, the animals were euthanized in 1 h intervals for a total period of 5 h, and the acyclovir content in plasma was analyzed using the high-performance liquid chromatography (HPLC) technique. During the experiment, each group of Wistar rats was kept in a polypropylene cage with clean wood shavings. The gavage was made in a separate room. The whole box was taken, and the mice received gavage one by one. At the end of the procedure, they returned to the vivarium to wait for the time interval between gavage and euthanasia. During this period, food and water remained available.

At the end of the determined period (1, 2, 3, 4, or 5 h), each rat was taken to an individual cage in a separate room, while the others remained in the vivarium. The anaesthesia, a mixture of ketamine and xylazine, was then administered. After administering anaesthesia and waiting for the appropriate time to complete the sedation of the animal, the euthanasia process began.

On a wooden board, the sedated animal was trapped by the legs with masking tape. With the aid of surgical scissors and forceps, the animal's trunk was opened to reveal a vena cava located below the liver. With the aid of a needle (0.70 × 30 mm) and a 5 mL syringe, 4 mL of blood was collected from the animal. In some cases, when it was not possible to collect this volume, some blood was collected from the heart. The blood was transferred to an ethylene diamine tetraacetate (EDTA) black tube, which was stored in a refrigerator. The blood samples collected in tubes (BD Vacutainer K2EDTA 7.2 mg, 4 mL) containing EDTA were rapidly centrifuged at 2880×*g* (4000 rpm) for 10 min. Then, the serum of each sample was separated and transferred into 1.5 mL polypropylene tubes and stored at − 20 °C until analysis.

### Analysis of Wistar rat blood based on Stulzer et al.^33^

The reagent and chemicals used were Acyclovir from Viafarma (SM emprendimentos farmaucéticos Ltda., São Paulo, Brazil). HPLC-grade (Chromasolv^®^) methanol and trifluoroacetic acid purchased from Sigma-Aldrich. Ultrapure water was prepared using a Milli-Q Academic water-purification system (Millipore, Milford, MA, USA).

Stock solutions of acyclovir (0.217 mg mL^−1^) were prepared by dissolving the drug in deionized Methanol: H_2_O (50:50, v/v) and stored in a refrigerator at 4 °C. For the calibration curve, the acyclovir stock solution was diluted with deionized water to obtain the different working solutions ranging from 20 to 2170 µg L^−1^.

Analysis of acyclovir solutions of the calibration curve and Wistar rat blood samples: a volume of 40 µL was injected into the HPLC system for analysis of 1.5 mL serum samples, after centrifugation (5 min at 6000×*g*) and being filtered in a 0.45 µm syringe filter (Chromafil^®^ Xtra Pa-45/25).

The concentration of acyclovir was measured by High-performance liquid chromatography (HPLC) system (Jasco). The HPLC system consisted of a UniverSil C18 analytical column (4.6 × 150 mm, 5.0 µm) with a fluorescence detector set at 260 nm (excitation) and 380 nm (emission). Chromatographic separation of acyclovir was achieved by a mobile phase comprising of Eluent A—0.08% [v/v] aqueous trifluoroacetic acid (pH = 2.30–2.35) and Eluent B- methanol 100%. The mobile phase was delivered at 1.5 mL min^−1^ and the gradient elution programs from eluents A and B were 0.00–7.00 min (96:4), 7.01–10.00 min (40:60), and 10.01–12.50 min (96:4). The column temperature was maintained at 25 °C during the analysis. The calibration curve was linear in the range of 20–2170 µg/L. The limit of quantification was defined as the lowest serum concentration of acyclovir quantified with a coefficient of variation of less than 20%.

### Ethical approval

The study is reported following ARRIVE guidelines (https://arriveguidelines.org). The approval for animal experiments was granted by “the ethics committee on the use of animals at Universidade Presbiteriana Mackenzie”. They approved the procedures of the referred project: CEUA 120/11/2014.

## Results and discussion

The several pseudoboehmite sample analysis showed that the pseudoboehmite synthesized is pure, without any other crystalline phases.

### Characterization of pseudoboehmite and the pseudoboehmite/acyclovir samples

The DTA and TG analysis of the pure pseudoboehmite indicated peaks characteristic of pseudoboehmite samples^34,35^. The sample has an endothermic peak at approximately 100 °C in DTA analysis due to the loss of water. Above 200 °C, mass loss is observed in the TG analysis, characteristic of pseudoboehmite transformation to gamma-alumina^36–39^, with a corresponding endothermic peak above 200 °C in the DTA analysis. This transformation finishes in the TG analysis at around 500 °C. Near 1200 °C, an exothermic peak is observed due to the last phase transformation of alumina, promoting the formation of alpha-alumina^15^.

**Figure 1:**
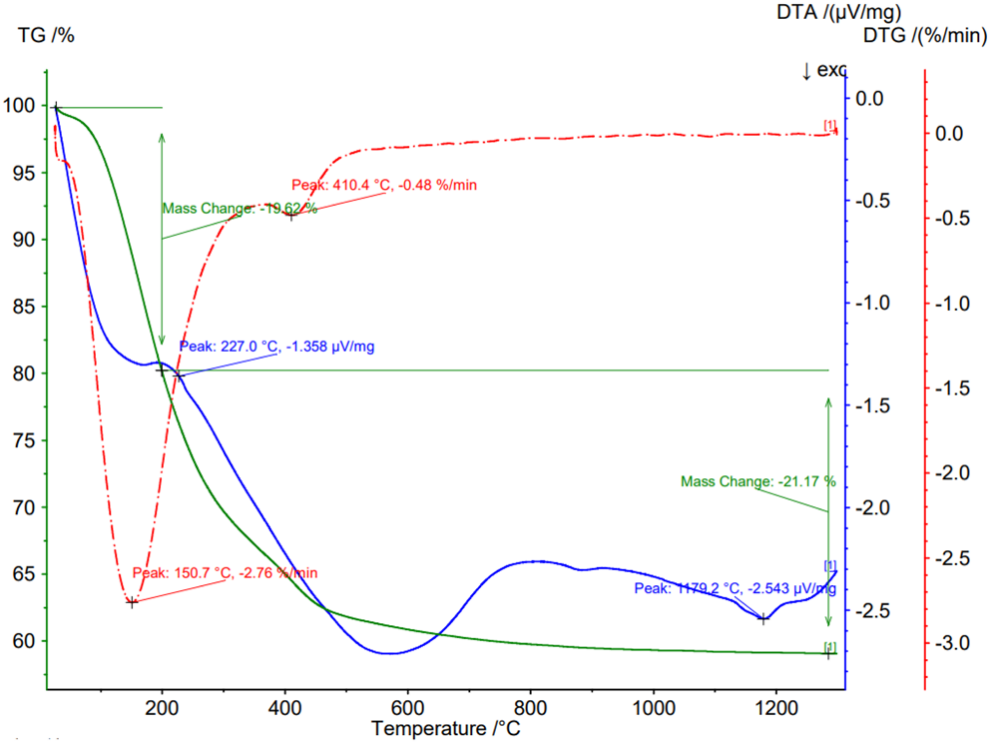
DTA and TG analysis of a pseudoboehmite sample containing acyclovir.

**Figure 2:**
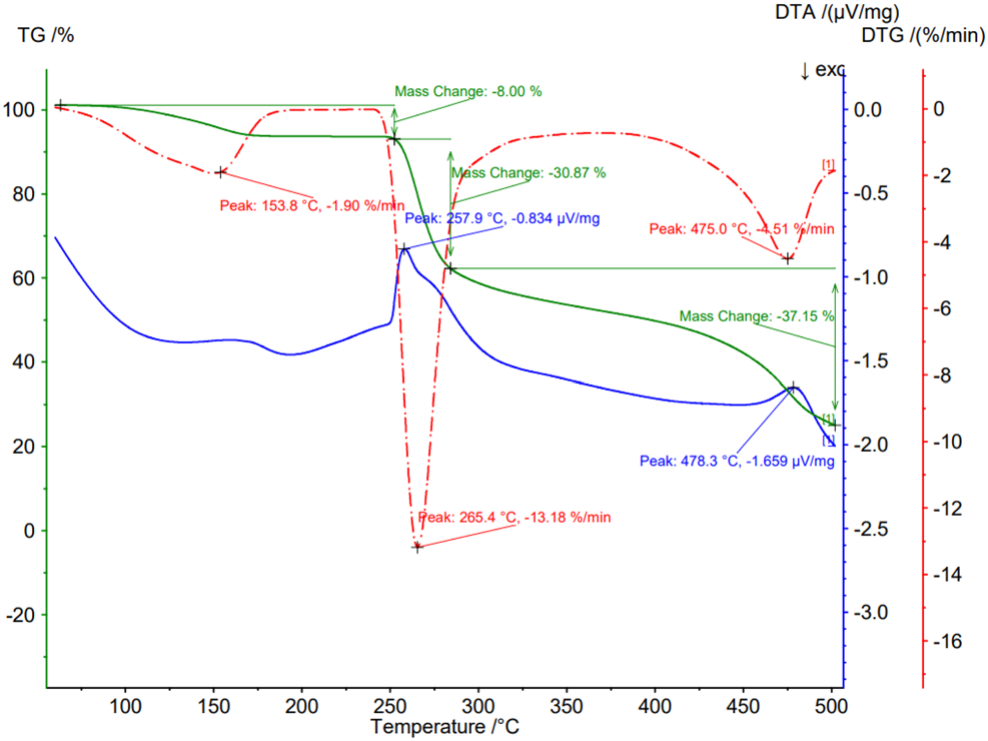
DTA and TG analysis of acyclovir.

The DTA and TG analysis of pseudoboehmite containing acyclovir (Fig. 1) shows no endothermic peak at 100 °C due to the release of adsorbed water. There is an endothermic peak in DTA (blue curve) due to the transformation of pseudoboehmite in gamma-alumina. The sharp melting peak of acyclovir at 257.9 °C (Fig. 2) was not observed in the pseudoboehmite/acyclovir sample (Fig. 1), due to the small amount of acyclovir in the sample. In Fig. 1, the last transformation of θ-alumina → α-alumina is observed at 1178 °C.

**Figure 3:**
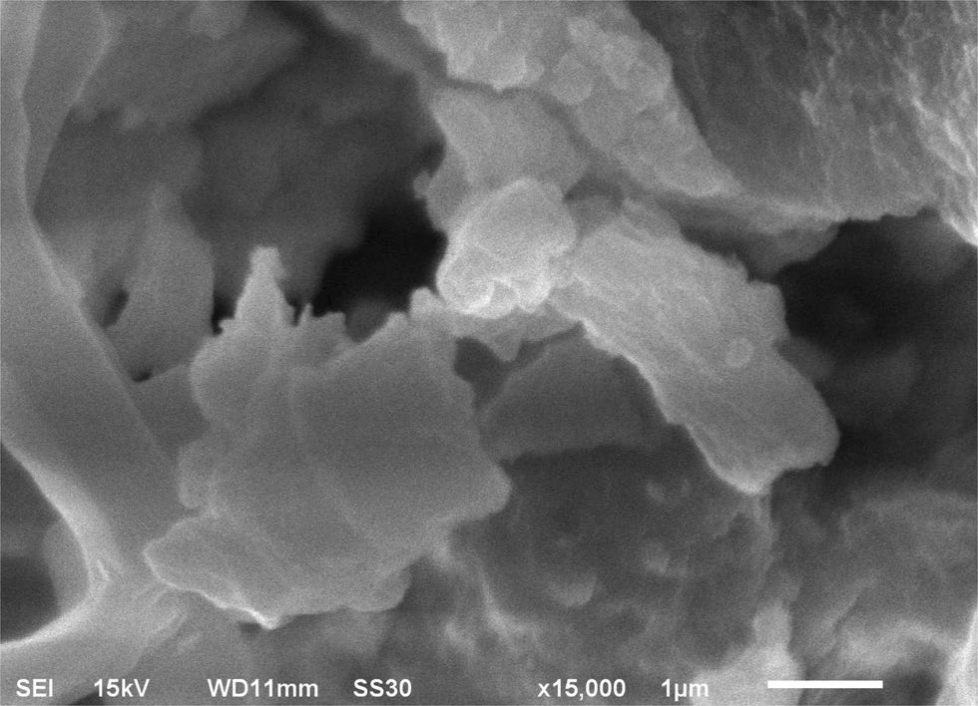
SEM image of a pseudoboehmite sample.

The SEM image of the pseudoboehmite sample (Fig. 3) revealed a porous material. Note that it is a freeze-dried sample. The drying of nanoparticles promotes particle agglomeration. The observed agglomerates show in the surface tiny plates. The EDS detector showed the presence of aluminium and oxygen in the sample. The semi-quantitative analysis of the elements using the EDS detector shows an atomic ratio of approximately two oxygen atoms to an aluminium atom, which is consistent with the pseudoboehmite chemical formula (AlOOH)n.

**Figure 4:**
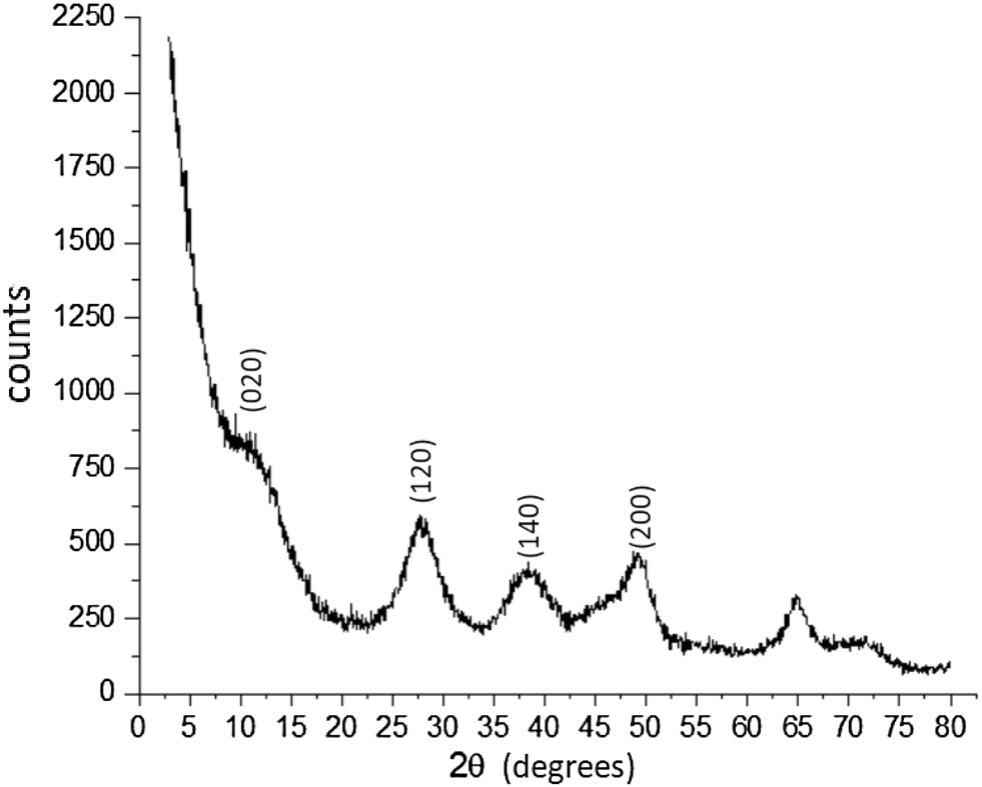
X-ray diffraction data of a pseudoboehmite sample.

The X-ray diffraction pattern of the pseudoboehmite sample (Fig. 4) shows the characteristic peaks of the pseudoboehmite structure according to the International center for diffraction data ICDD 00-021-1307 card and the literature^13,40–43^.

Instead of sharp peaks typical of a crystalline solid, a characteristically weak diffraction intensity is observed with a pronounced line broadening of the peaks due to small crystallite sizes and a higher d(020) value in the X-ray diffraction data^44–46^.

**Figure 5:**
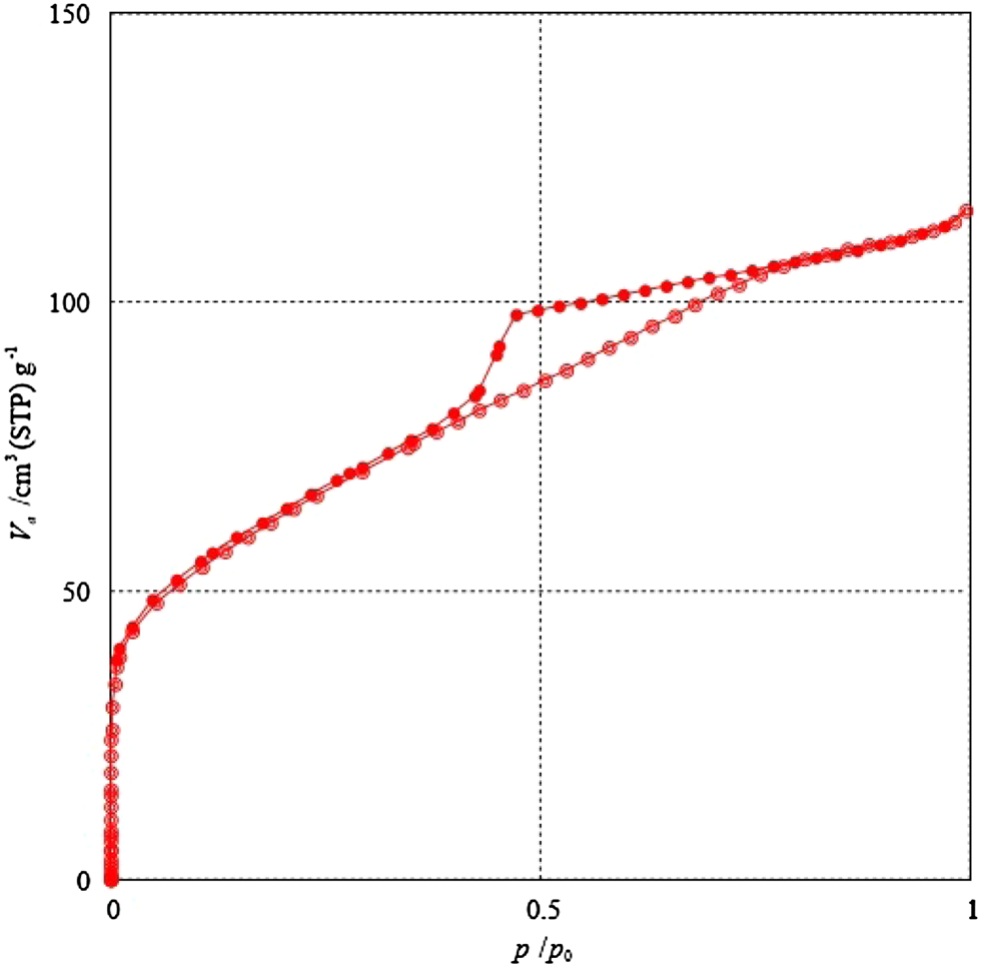
N_2_ adsorption isotherm of pseudoboehmite sample.

The nitrogen adsorption isotherm of the pseudoboehmite sample (Fig. 5) shows the characteristic type IV isotherm^47^. This indicates that the pseudoboehmite sample contains mesoporous and macropores. At the beginning of adsorption, near p/p_o_ = 0, the isotherm shows a line nearly parallel to the y-axis. This is characteristic of the presence of micropores in the sample. The specific surface area determined from the BET method is 218 m^2^ g^−1^.

The nitrogen isotherm has a hysteresis with an H1^47^ shape which is often associated with porous materials known, from other evidence, to consist of agglomerates or compacts of approximately uniform spheres in a regular array. The materials with H1 hysteresis have narrow distributions of pore size.

**Figure 6:**
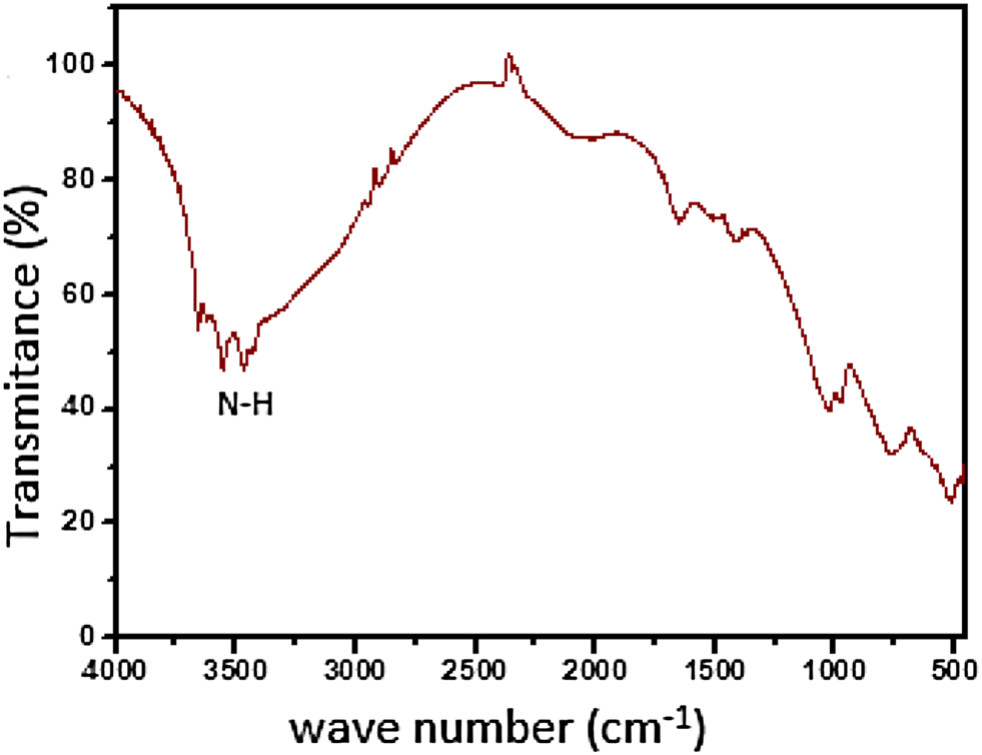
FTIR spectrum of a pseudoboehmite/acyclovir sample.

**Figure 7:**
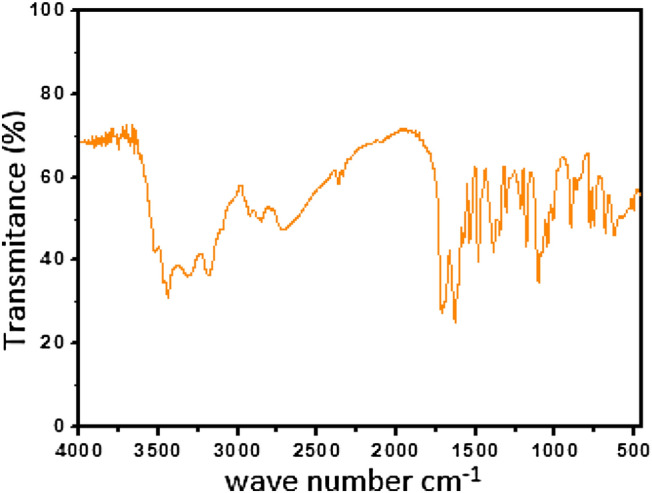
FTIR spectrum of acyclovir sample.

The pseudoboehmite synthesis was realized at room temperature and the sample was not aged. Well defined crystals are formed only at the higher synthesis temperature. The FT-IR spectra of the pseudoboehmite sample show a very strong and broadband centred at approximately 3400 cm^−1^ with no shoulders. This is characteristic of not well-crystallized pseudoboehmite^48^. The pseudoboehmite/acyclovir sample (Fig. 6) contains well-defined bands in the near 3500 cm^−1^. The bands at 3438 and 3516 cm^−1^ refer to the symmetric and asymmetric axial deformations of the N–H present in the acyclovir sample (Fig. 7)^49^.

**Figure 8:**
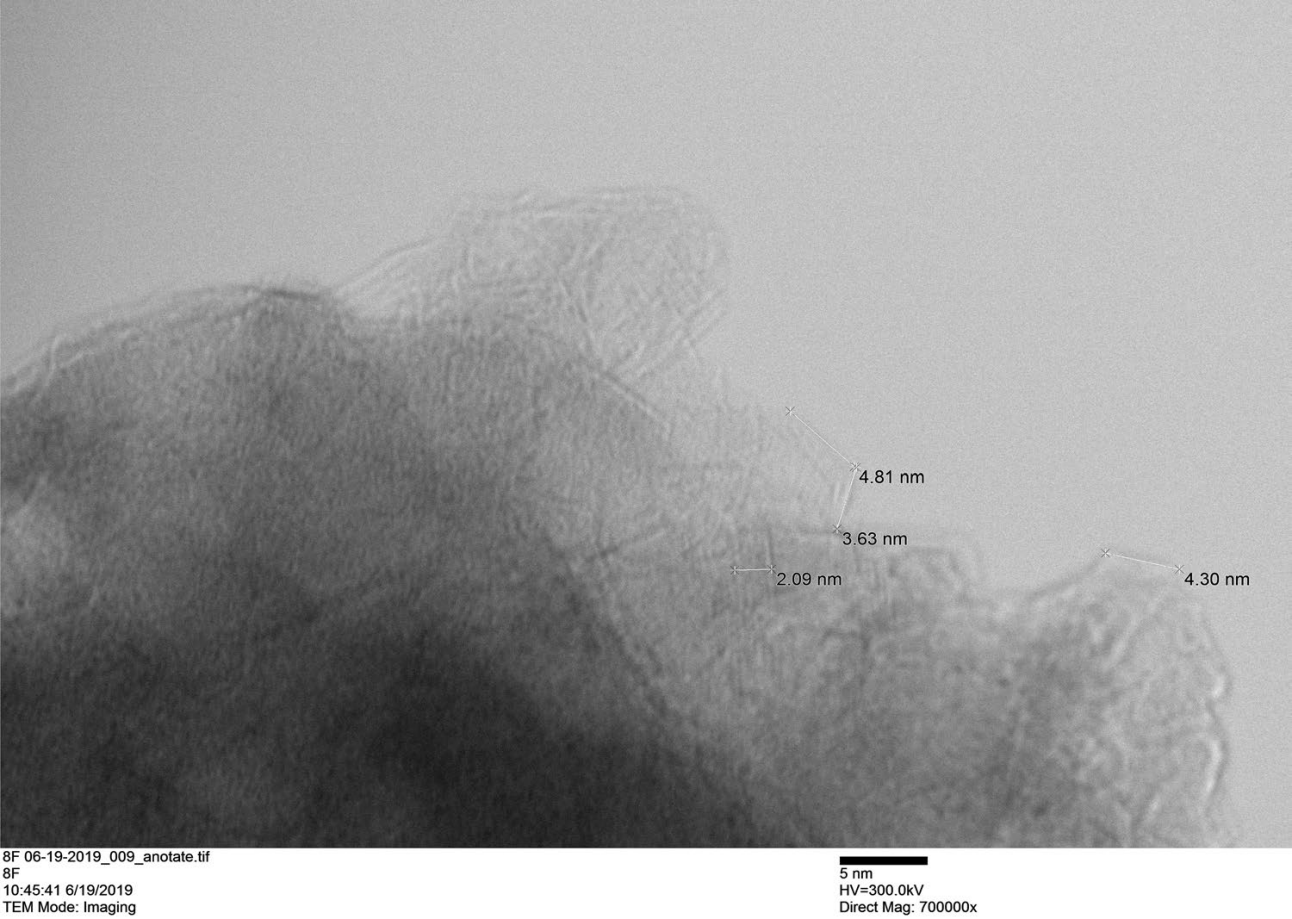
TEM image of an aggregate of pseudoboehmite nanoparticles.

Figure 8 shows the TEM image of a pseudoboehmite sample at the edge of an aggregate of a pseudoboehmite nanoparticle. The particles, consisting of tiny plates in the range of colloidal dimensions, are anisometric, with a lateral dimension less than 5 nm. At the edges of the aggregate where the individualized particles are observed, the particle and the region immediately adjacent (void) have a very close intensity contrast, showing that the particles are extremely thin (probably < 1 nm). The results are similar to the TEM results previously reported in the literature^48,50,51^. Magela de Aguilar Cruz (1998) reported well-defined hexagonal crystals^51^.

**Figure 9:**
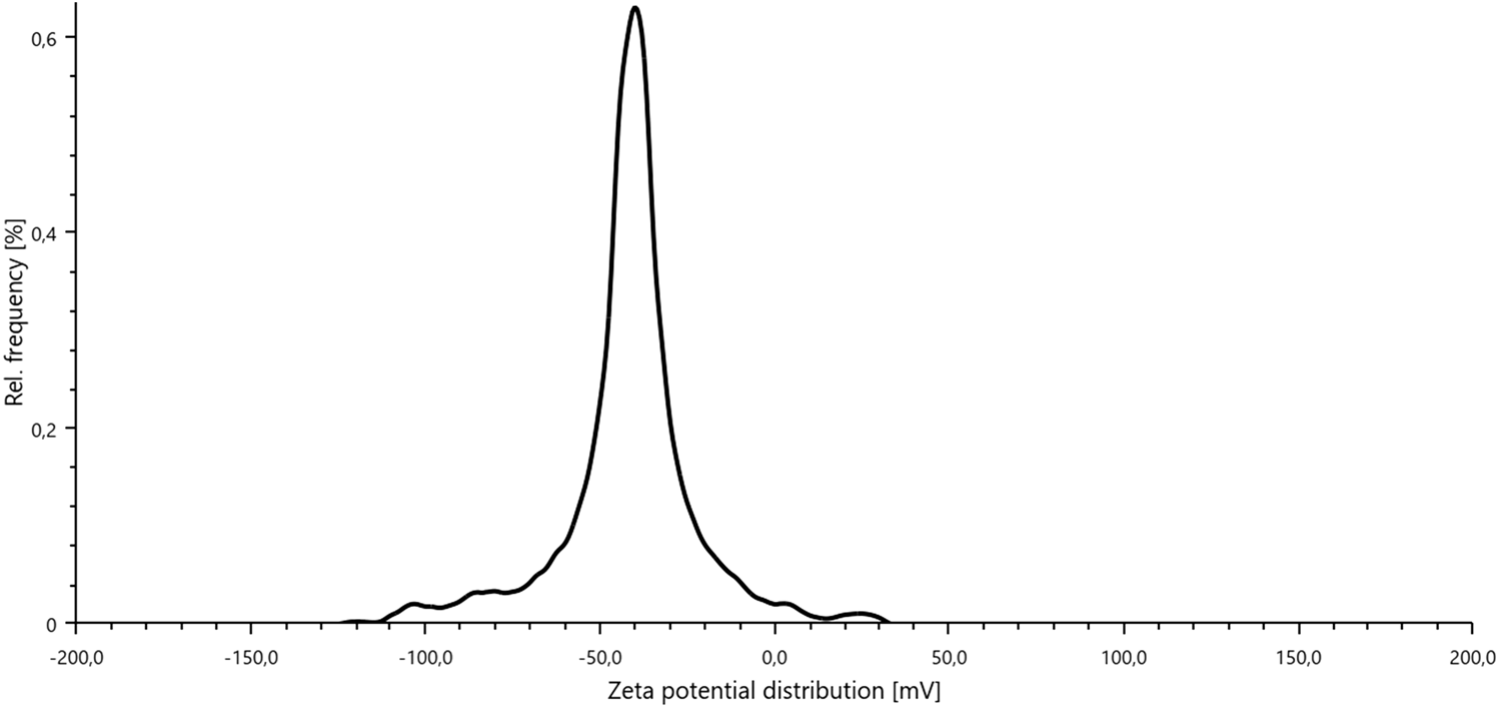
Zeta potential of pseudoboehmite gel.

The zeta potential obtained for the gel was -42.6 mV (Fig. 9). The higher the zeta potential, the higher the repulsion among the particles, and the more stable the system. For zeta potential in this range, it is possible to affirm that the pseudoboehmite gel is a stable dispersion^52^.

### Toxicity results of pseudoboehmite

**Figure 10:**
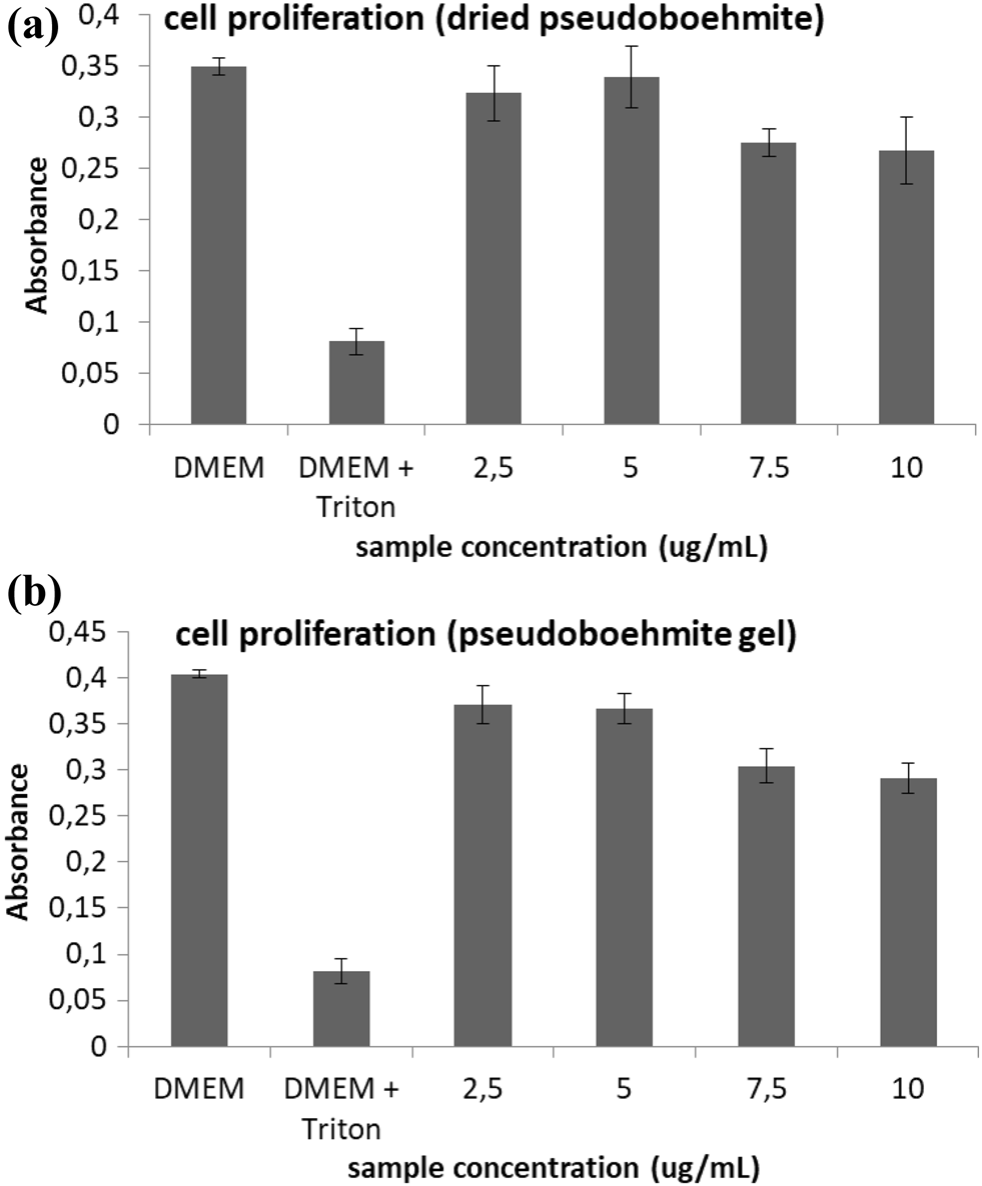
Results of pseudoboehmite gel toxicity using Caco-2 cell line (ATCC) for the (**a**) dried sample and the (**b**) pseudoboehmite gel.

Our results suggest low toxicity up to a concentration of 10 mg/mL, in gel and the dried pseudoboehmite nanoparticles. Figure 10a summarizes the results for the dried pseudoboehmite sample and Fig. 10b shows the results for the gel pseudoboehmite. For the two samples of pseudoboehmite, the cell proliferation was not.

### Analysis of acyclovir calibration curve

**Figure 11:**
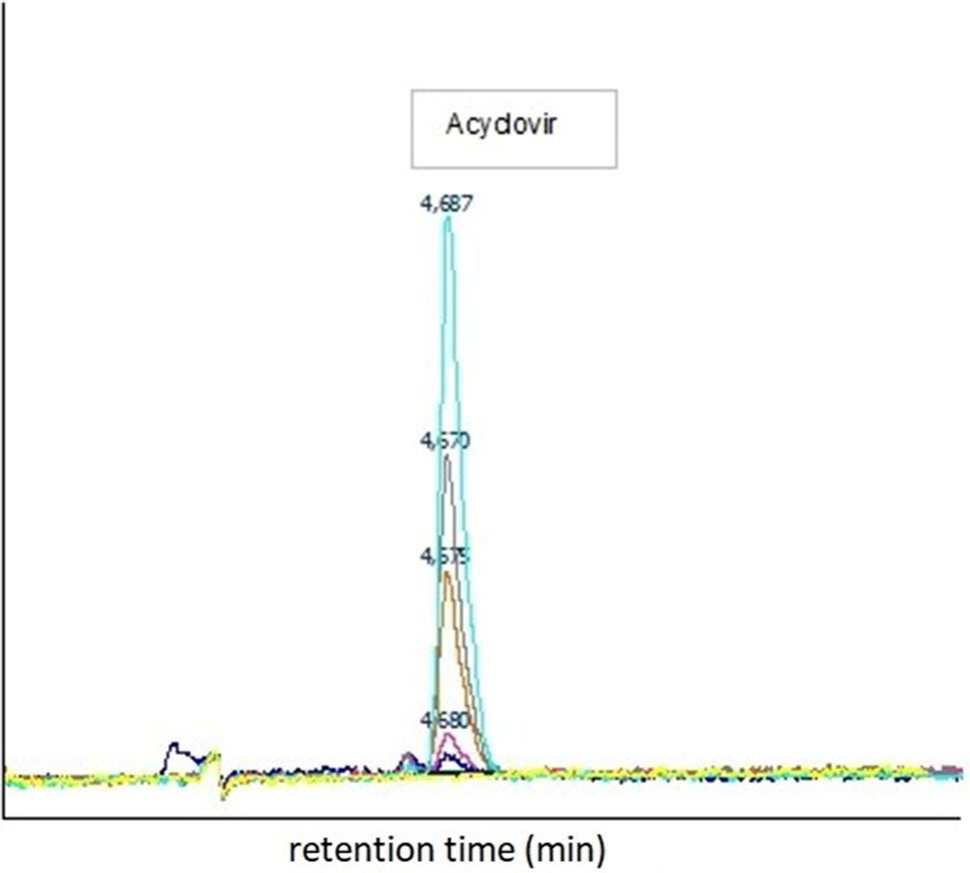
Typical chromatograms of acyclovir in different concentrations (22, 108, 542, 1084 and 1626 µg/L).

**Figure 12:**
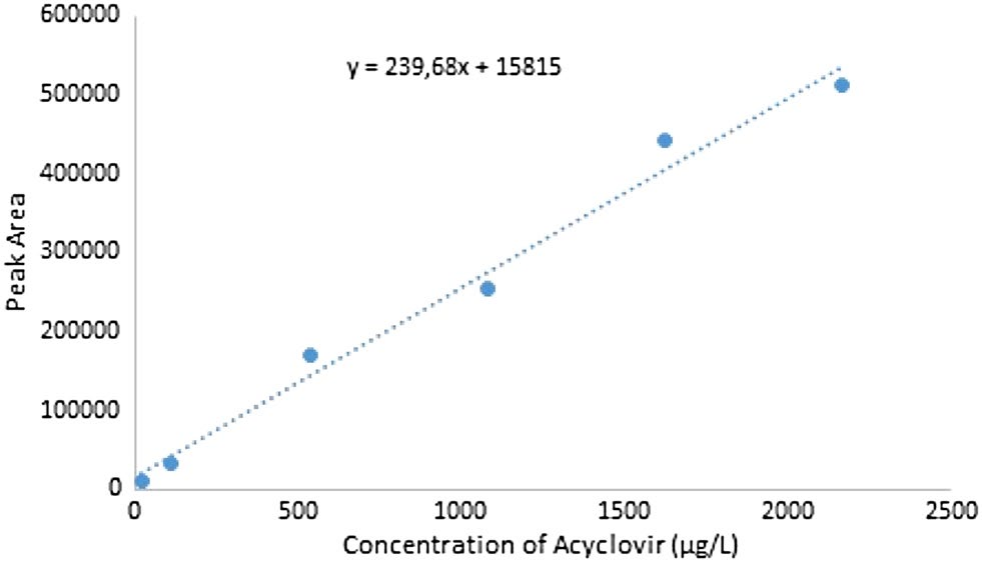
Calibration curve of acyclovir.

Typical chromatograms of acyclovir in different concentrations and the calibration curves are shown in Figs. 11 and 12, respectively.

The results of the calibration analysis show a very good adjustment in Fig. 12 for the linear equation y = 239.68x + 15.815, where y is equal to the peak area and x is equal to the acyclovir concentration. R^2^ is equal to 0.9851.

### Analysis of acyclovir in Wistar rat blood

**Figure 13:**
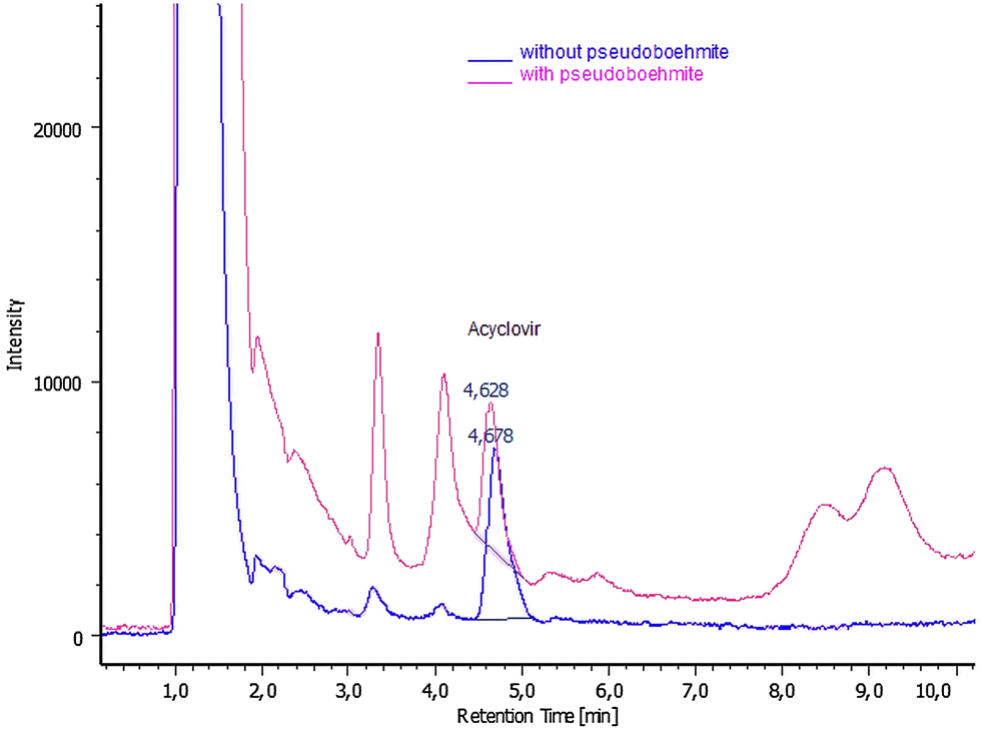
Chromatograms of serum samples of Wistar rats treated without (blue) and with pseudoboehmite (magenta).

The retention time of acyclovir was around 4.7 min. The present method has been applied to compare the effect of acyclovir in the blood of the Wistar rats treated with and without pseudoboehmite. Typical chromatograms of serum samples, without and with pseudoboehmite, are shown in Fig. 13. It is observed the evidence of the drug in the samples with a retention time of 4.7 min. characteristic of acyclovir.

**Figure 14:**
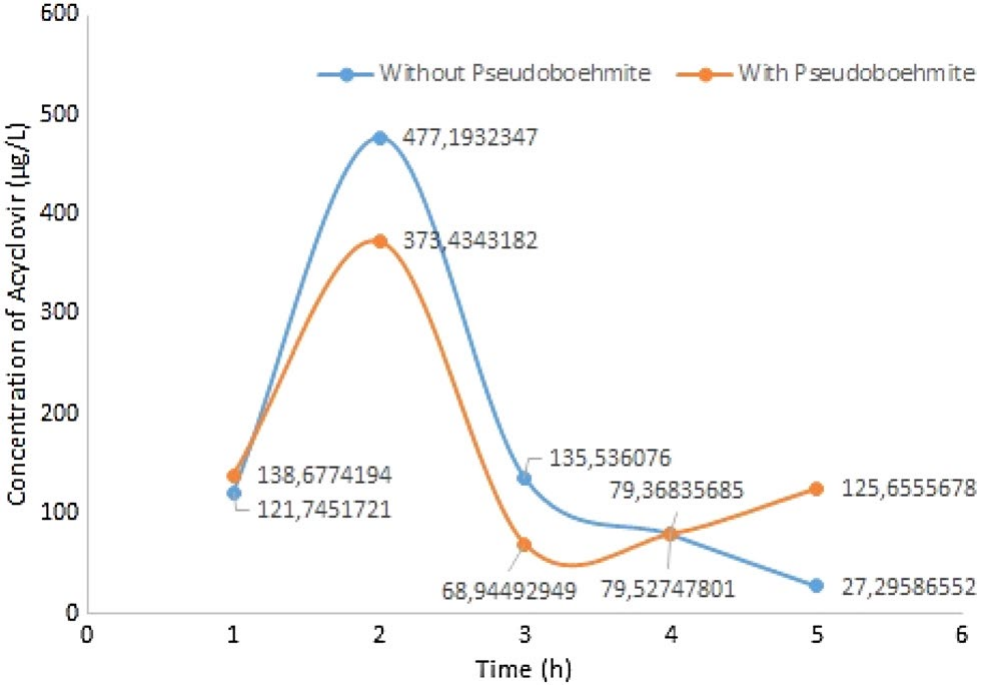
Acyclovir concentration in the blood of Wistar rats. Blue is without pseudoboehmite and orange is with pseudoboehmite.

Figure 14 shows the release of acyclovir with and without pseudoboehmite. Each data point provided in the graph of Fig. 14 corresponds to the mean of five trials. The pharmacokinetic profile of acyclovir is similar for both administrations. It is observed that in the Wistar rats treated with pseudoboehmite and acyclovir, the acyclovir content increases after 1 h of administration reaching a peak after ~ 2 h. The result is similar to the data reported in the literature^23^. However, in the first 3 h, the content of acyclovir was higher in the blood of the Wistar rats in which the drug was not administered with the pseudoboehmite. For the same group of Wistar rats after about 3.5 h, there is a new growth of acyclovir content ending at 125.7 µg/L after 5 h of administration. This improvement in bioavailability after 5 h could be due to the acyclovir adsorbed in the pseudoboehmite that was released later. The release from the nanoparticles is governed by the solubility of the drug, its diffusion, and the desorption of drugs through the pseudoboehmite nanoparticles.

For mice that were treated only with water and acyclovir, the behaviour is similar to the release in the presence of pseudoboehmite. However, after reaching a peak of around 2 h, the acyclovir content decreases continuously, reaching a concentration of 27.3 µg/L after 5 h of administration.

## Conclusions

Our results revealed that the synthesized pseudoboehmite is porous, exhibiting macropores, mesopores, and micropores. We verified the presence of nanoparticles in the pseudoboehmite samples using infrared spectroscopy, transmission electron microscopy, and X-ray diffraction techniques and by the hysteresis of nitrogen isotherm. The HPLC data shows that it was adequate for acyclovir analysis in the plasma of Wistar rats.

The toxicity results show that pseudoboehmite is safe up to a concentration of 10 mg/mL, in gel and the dried pseudoboehmite nanoparticles.

Administration of acyclovir by gavage with pseudoboehmite in Wistar rats, when compared to the administration of acyclovir in water by gavage, showed that the content of acyclovir in the plasma of rats increases after about 2 h of administration in rats that were administrated with acyclovir and pseudoboehmite/acyclovir. The release profile is very similar to both conditions.

For the tests using pseudoboehmite, the medium of acyclovir content in the plasma of Wistar rats after 5 h administration is 4.6 times higher than the acyclovir content of the animals that were administered with acyclovir in water only. The in vivo drug release results obtained in this study shows the potential of acyclovir loaded pseudoboehmite nanoparticles as a drug delivery system.

Future experiments with acyclovir loaded in pseudoboehmite using longer periods of time will determine the concentration of acyclovir in Wistar blood after 5 h administration.
